# Artificial Intelligence for Cardiovascular Health in China: Landscape and Challenges

**DOI:** 10.1016/j.jacasi.2026.04.019

**Published:** 2026-06-06

**Authors:** Hangkuan Liu, Linjie Li, Xin Zhou, Qing Yang

**Affiliations:** Department of Cardiology, Tianjin Medical University General Hospital, Tianjin, China

**Keywords:** artificial intelligence, cardiovascular health, China, personalized medicine

**Figure:**
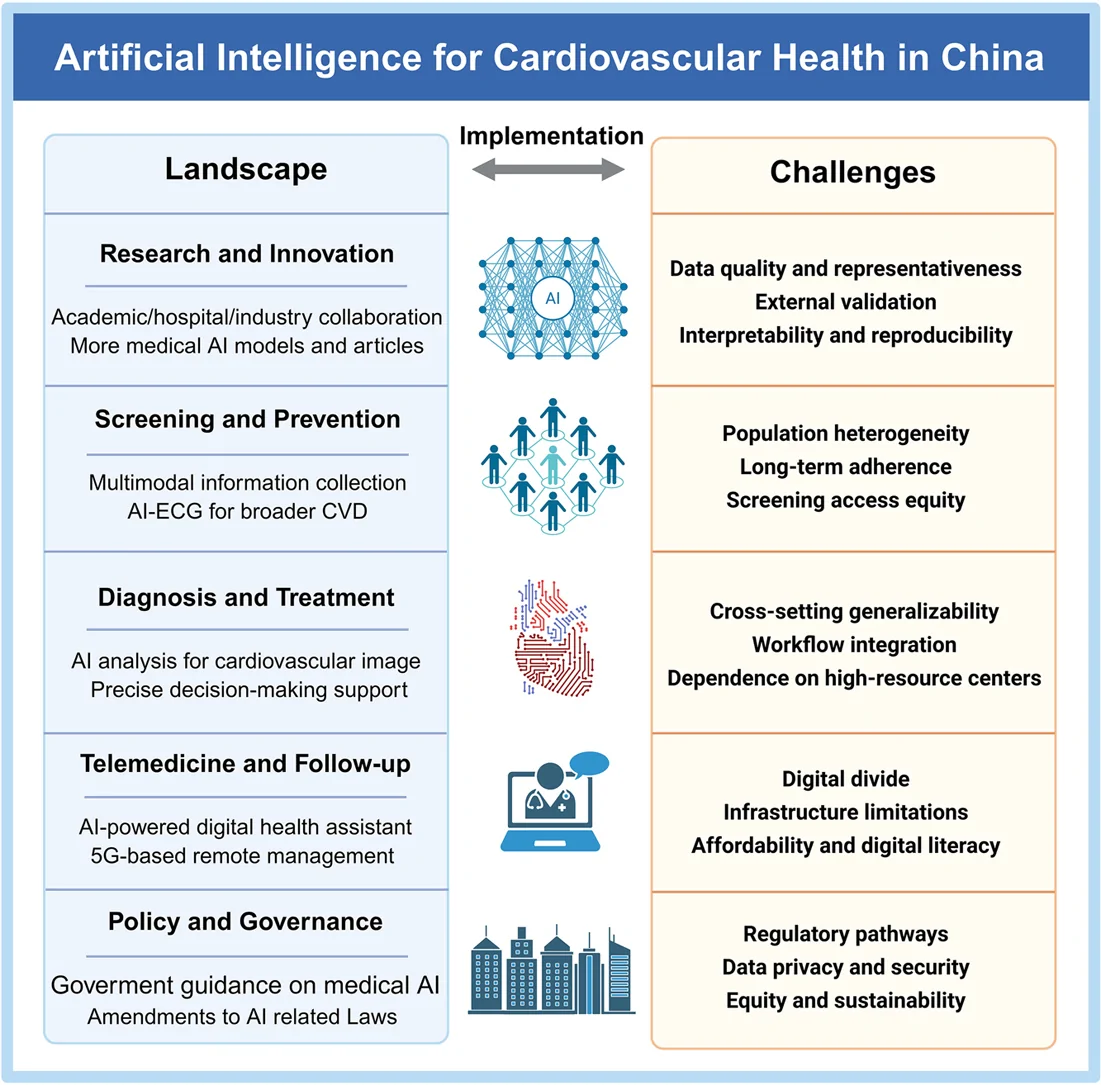

Cardiovascular disease (CVD) burden in China is mainly driven by the large population and rapid population aging. As of 2021, China’s CVD mortality rate (357.4 per 100,000 persons) is higher than the global average (258.8 per 100,000 persons) and contributed to a 103.7% increase in absolute deaths over the past 3 decades, marked by disparities across sexes and provinces.^1^ Simultaneously, China has made advancements in the medical artificial intelligence (AI) field, evidenced by a growing number of AI-related medical publications and early clinical applications.^2^ However, despite rapid improvements in medical AI performance, its clinical translation in cardiovascular care remains at an exploration stage.^3^ The gap between AI model performance and real-world implementation is particularly pronounced in China, which has a large and aging population, marked regional disparities, and a tiered health care system. The Chinese Academy of Sciences and major universities with top-tier affiliated hospitals in China play a pivotal role in bridging the gap between technical innovation and clinical research in cardiovascular health.^4^ To inform researchers and health care providers the advance knowledge about AI-enable CVD management, we summarize the landscape and challenges of AI for cardiovascular health in China (Figure 1).

**Figure 1:**
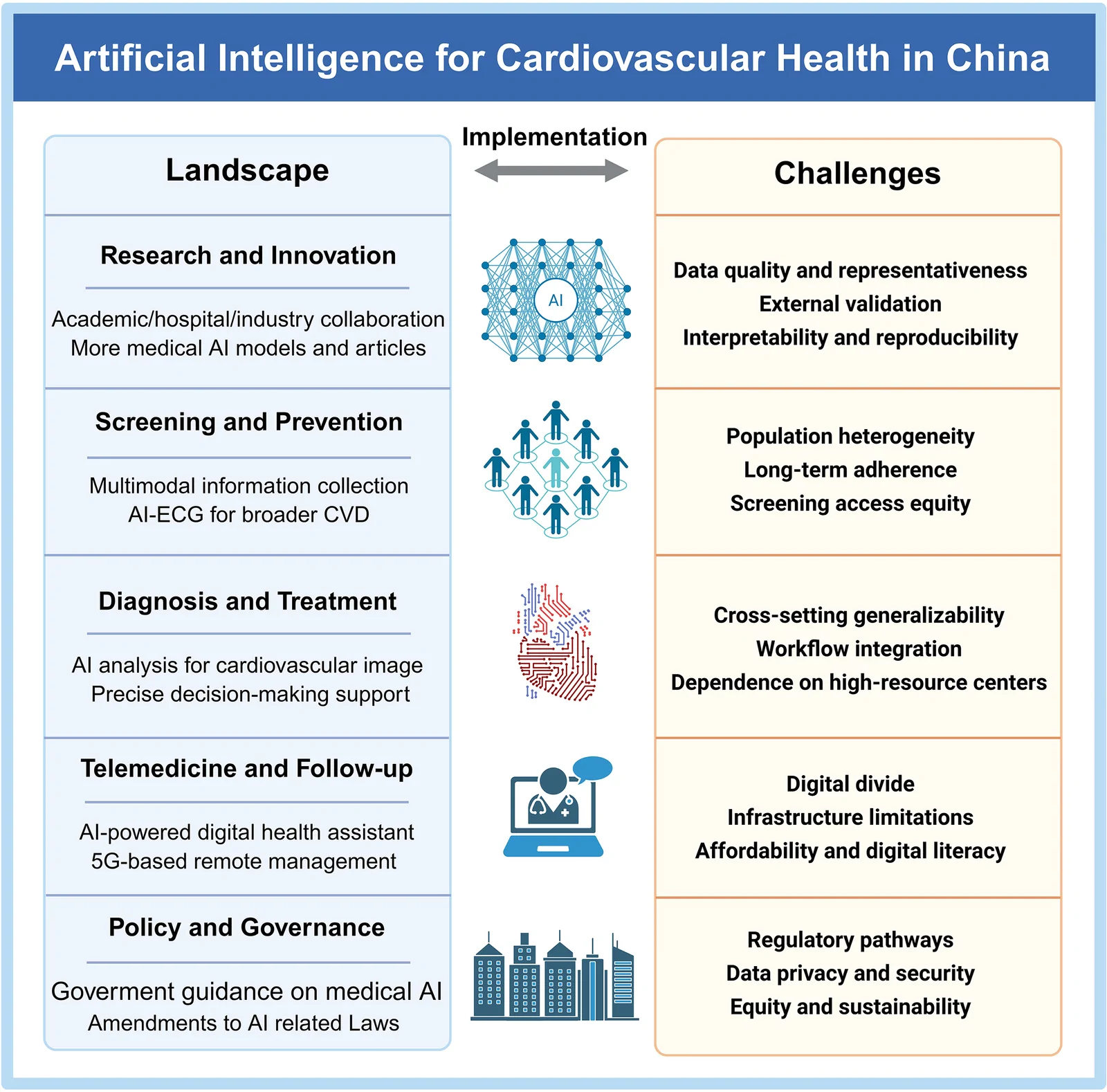
Landscape and Challenges of AI for Cardiovascular Health in China This figure illustrates the current landscape of artificial intelligence (AI) for cardiovascular health in China and the major challenges to implementation across 5 domains: research and innovation, screening and prevention, diagnosis and treatment, telemedicine and follow-up, and policy and governance. The figure is created in https://BioRender.com. CVD = cardiovascular disease; ECG = electrocardiography.

## Landscape of AI for Cardiovascular Health in China

AI-enabled tools are increasingly being introduced into cardiovascular care in China, spanning research and innovation, screening and prevention, diagnosis and treatment, telemedicine and follow-up, under the national policy and governance. In total, medical AI in China is moving beyond technical proof-of-concept toward broader clinical translation for CVD management.

### Research and innovation

Cardiovascular AI research in China has expanded rapidly, supported by collaborations among academic institutions, tertiary hospitals, and industry.^2^ As of the middle of 2025, China ranked second globally in publication output related to AI and CVD (372 articles), behind the United States (432 articles).^5^ Current efforts extend from model development based on electronic health records, imaging, and wearable data to early translational studies in clinical decision support and remote care. This research ecosystem has provided an important foundation for the wider application of AI in cardiovascular medicine, although much of the evidence remains concentrated in top health care centers and early-stage validation.

### Screening and prevention

Traditional CVD risk prediction models rely largely on limited, cross-sectional clinical indicators. In principle, AI technologies can integrate large-scale, multimodal, and dynamic data from electronic health records, medical imaging, wearable devices, and multi-omics platforms, thereby improving risk stratification and early disease detection.^6^^,^^7^ However, broad clinical implementation of most AI-enabled tools remains limited in China. Among currently available approaches, AI-enabled electrocardiography (ECG) stands out as a scalable and relatively cost-effective screening tool. To predict valvular heart disease by ECG data, Liang et al^8^ from Fudan University developed an AI-ECG model based on residual convolutional neural network integrated with discrete-time survival loss functions. In internal validation, the AI-ECG model showed moderate performance for predicting mitral regurgitation (C-index, 0.774), tricuspid regurgitation (C-index, 0.691), and aortic regurgitation (C-index, 0.793). In external validation, the model also performed reasonably well in outpatients from Beth Israel Deaconess Medical Center, with corresponding C-indices of 0.755, 0.665, and 0.765, respectively.^8^ This study reflects AI-ECG capability to capture signals potentially associated with subclinical chamber remodeling. Although this AI-enabled tool has so far been validated only in selected populations, it illustrates how the convergence of AI and ECG data may expand the clinical utility of ECG beyond the traditional manual diagnosis of arrhythmias and myocardial infarction, with potential value as a relatively low-cost and scalable approach to broader CVD screening and prevention.

### Diagnosis and treatment

Furthermore, AI enhances the interpretation of various cardiovascular images, unlocking a wealth of data largely underutilized by cardiologists. Leveraging AI tools enables the detection of subtle phenotypic variations and the establishment of reproducible, standardized workflows. A recent study led by Peking Union Medical College Fuwai Hospital in collaboration with multiple external partners has developed automated cardiac magnetic resonance-based interpretation systems capable of screening and diagnosing 11 types of CVD, including ischemic heart disease, pulmonary hypertension, and congenital heart disease.^9^ This novel AI-assisted system explores more information from cardiovascular images and improves the predictive accuracy, interpretation efficiency, and scalability for various CVDs, thereby alleviating the clinical workload.

Based on accessibility, AI enhances the quality of cardiovascular care in primary health care institutions. Targeting underserved urban populations, the LIGHT (Learning Implementation of Guideline-based Decision Support System for Hypertension Treatment) study from Peking Union Medical College Fuwai Hospital demonstrates that using the clinical decision support system in primary care in urban regions of China improved the provision of guideline-accordant antihypertensive treatment.^10^ Compared with usual care, AI-driven clinical decision support system application led to a modest reduction in blood pressure (−1.5 mm Hg vs 0.3 mm Hg, *P* = 0.006).^10^ Although this study suggests that AI-assisted clinical decision support may improve the standardization of antihypertensive care, its broader applicability to rural and underresourced settings in China remains to be established. Moreover, the clinical significance of modest blood pressure reduction, as well as its implications for long-term CVD outcomes, and cost-effectiveness, needs further evaluation.

In the areas of CVD invasive intervention, AI also enables precise, noninvasive functional assessment. Angiography-derived fractional flow reserve is an innovative AI-driven approach for evaluating coronary artery lesions by estimating contrast flow velocity, which is applied in precise percutaneous coronary intervention in China. Recently, the FLAVOUR II (Angiography-Derived Fractional Flow Reserve vs Intravascular Ultrasound to Guide Percutaneous Coronary Intervention in Patients With Coronary Artery Disease) study led by Hu et al^11^ from Zhejiang University demonstrates that angiography-derived fractional flow reserve-guided comprehensive percutaneous coronary intervention strategy (including revascularization decision making and stent optimization) was noninferior to intravascular ultrasound guidance. AI-assists the precise decision-making support for CVD management.

### Telemedicine and follow-up

The cornerstone of primary and secondary prevention of CVD lies in accessible medical resources and comprehensive management, including dynamic monitoring, lifestyle management, pharmacotherapy, and invasive intervention. In China, AI technology provides opportunities to reduce the gap in medical inequality between urban and rural areas. In 2024, Zhejiang became the first province in China to launch the province-wide AI-powered digital health assistant (*Anzhener*), which can provide cloud-based medical consultation services accessible to residents.^12^ This AI-assisted telemedicine pattern may provide accessible health services nationwide in China.

Meanwhile, China's rapid deployment of 5G infrastructure provides the critical backbone for scaling AI-enabled cardiovascular solutions.^13^ By leveraging ultralow latency, high bandwidth, and seamless connectivity, 5G technology accelerates the real-time transmission and analysis of multimodal data across patients, medical devices, and hospital information systems. This ecosystem is essential for realizing advanced telemedicine scenarios, including remote patient monitoring, cloud-based consultation, and remote robotic-assisted cardiovascular interventions. Crucially, by mitigating connectivity constraints, 5G enables the seamless expansion of successful AI pilots from urban centers to remote and underserved regions, particularly in Western China.^13^ The integration of 5G-enabled, AI-powered tools may help reduce regional disparities and facilitate access to real-time, expert-level cardiovascular care at the grassroots level, thereby extending the reach and utility of high-quality medical resources.

### Policy and governance

In 2024, China’s National Health Commission released a reference guide on the application scenarios of AI tools across various health areas.^14^ This guideline serves as a strategic framework to leverage AI for enhancing quality, efficiency, and equity of health care delivery, which is important in cardiovascular health. The core clinical scenario about AI application in CVD from China’s perspective includes primary public health services (health screening, health education), medical service management (medical services, pharmaceutical services, hospital management, traditional Chinese medicine), health industry development (medical robotics, drug discovery and development), and medical education and research (medical education, medical research).^14^ Meanwhile, AI-related legal and regulatory frameworks in China are being progressively improved. In January 2026, amendments to China’s Cybersecurity Law formally entered into force, extending its relevance to aspects of AI governance. Together with the Data Security Law and the Personal Information Protection Law, the amended law forms a key part of China’s data and cybersecurity framework, with important implications for balancing security, compliance, and innovation in medical AI applications.^15^ This AI-related policy and governance may enhance both cost-effectiveness and the utilization of health care resources across the spectrum of CVD management in China.

## Challenges for AI in Cardiovascular Care in China

AI integration is reshaping CVD management by bridging the gap between population-level insights and individualized care for heterogeneous patients. Despite strong national strategies like “Healthy China 2030” and the unique advantage of vast real-world health data in China,^16^ the nationwide application of AI in cardiovascular health faces various challenges.

### Research and innovation

Given the rapid evolution of medical AI, the central challenge for cardiovascular AI research in China is no longer simply to produce additional CVD predictive models or publications,^5^ but to generate clinically meaningful evidence based on established clinical AI reporting standards (such as CONSORT-AI, SPIRIT-AI, or TRIPOD+AI),^17^ high-quality, representative, and interoperable data, supported by rigorous external validation, interpretability, and reproducibility. These issues are particularly important in China, where substantial differences exist across provinces, hospital tiers, and patient populations, creating major barriers to data interoperability and limiting the generalizability of findings derived from single-center datasets. Therefore, systematic data standardization and coordinated integration of AI into national cohorts and research platforms are essential for advancing cardiovascular research and supporting broader application across regions in China.^18^

### Screening and prevention

Although AI-enabled tools have shown promise for cardiovascular screening and prevention in China, broader implementation remains constrained by 3 interrelated barriers: population heterogeneity, long-term adherence, and equitable access to screening tools. These barriers are particularly important because demographic profiles, cardiovascular risk patterns, digital readiness, and health care resources vary substantially across regions and population groups. Meanwhile, primary care institutions, particularly in rural and underresourced regions in China, often have limited access to routine screening tools and fewer opportunities to adopt AI-enabled preventive strategies. As a result, tools developed in a one-size-fits-all manner may not perform equally well or achieve the same uptake across diverse settings.^19^ The future value of AI in cardiovascular prevention depends not only on technical performance, but also on whether such tools can be adapted to heterogeneous populations, sustained over time, and deployed in ways that improve access rather than reinforce existing disparities.

### Diagnosis and treatment

In diagnosis and treatment support, China has developed promising AI applications in ECG interpretation, cardiovascular imaging, and clinical decision support.^8^, ^9^, ^10^, ^11^ However, broader AI implementation is limited by cross-setting generalizability, workflow integration, and continued dependence on high-resource centers. These barriers are particularly relevant in China’s tiered health care system, where differences in equipment, data quality, specialist expertise, and care pathways may substantially affect model performance and clinical usability outside tertiary hospitals.^20^

### Telemedicine and follow-up

Telemedicine and AI-assisted follow-up may offer the greatest opportunity to reduce geographic disparities in cardiovascular care in China, especially for rural and underresourced settings. At the same time, this domain faces some of the most visible implementation barriers, including the digital divide, infrastructure limitations, affordability, and variable digital literacy among patients and providers.^21^ In China, the populations most likely to benefit from remote cardiovascular care are often those with the weakest connectivity, the lowest digital readiness, and the greatest difficulty sustaining device-based follow-up.^22^ Overcoming the gap needs the strong support from government or associations.

### Policy and governance

China’s strong policy support has created an important foundation for AI-enabled cardiovascular care, but supportive strategy alone is not sufficient for implementation at scale. Key barriers remain in regulatory pathways, data privacy and security, equity, and sustainability of funds and facilities, particularly for medical AI tools, which require continuous updating, maintenance, and postmarket monitoring.^23^

In China, the next stage of policy development will need to move beyond support of AI innovation toward clearer regulatory standards, accountable governance, and financing mechanisms that support equitable and sustainable deployment.^24^ Although risks of bias and unequal access remain, well-designed implementation under appropriate policy support and regulatory guidance may help prevent AI from reinforcing existing disparities and contribute to reducing systemic inequities in cardiovascular care.

## Conclusions

Continuous breakthroughs in medical AI are optimizing CVD management in China, shifting the traditional paradigm toward accessible and personalized cardiovascular health. China’s experience illustrates both the promise of AI in a large and rapidly digitalizing health system and the challenges of translating technical advances into equitable, scalable, and sustainable clinical practice. The experience in China may also provide useful lessons for other health systems implementing AI in cardiovascular care.

## Funding Support and Author Disclosures

This work was supported by the National Natural Science Foundation of China (82320108001, 82270349 from Dr Yang), Tianjin Key Medical Discipline (Specialty) Construction Project (TJYXZDXK-069C from Dr Yang), and Double First-Class Project of Tianjin Medical University (SYL002-303078100820, SYL002-303078100821 from Dr Yang). All other authors have reported that they have no relationships relevant to the contents of this paper to disclose.
